# Transnational HIV-AIDS Action and Citizen Diplomacy in the Late Soviet Union, 1988–1991

**DOI:** 10.1353/sex.00019

**Published:** 2025-05

**Authors:** Siobhán Hearne

## Introduction

In July 1991, as George H. W. Bush and Mikhail Gorbachev met in Moscow to sign the Strategic Arms Reduction Treaty, sixty-nine North American lesbian and gay activists arrived in Leningrad to participate in the International Gay and Lesbian Symposium and Film Festival.^1^ The Symposium was the first event of its kind to be held in the USSR. Across ten days, participants attended workshops, discussion sessions, film screenings, and demonstrations in both Leningrad and Moscow. At one Leningrad session dedicated to discussing AIDS, an HIV-positive delegate from the USA was giving a presentation about his diagnosis when Soviet gay activist Roman Kalinin spontaneously pushed a man called Gennady Roshchupkin onto the stage.^2^ At that moment, Roshchupkin disclosed his HIV-positive status, becoming the first Soviet citizen to do so publicly in the presence of journalists.^3^

The International Symposium was just one of many examples of the transnational mobilization of Soviets and North Americans around the issues of HIV and AIDS in the final years of the Soviet Union. The HIV-AIDS epidemic unfolded at a time when the USSR was undergoing profound changes to its social, cultural, economic, and political fabric. The reforms implemented under the premiership of Mikhail Gorbachev (1985–1991) resulted in widespread civic mobilization and increased contact between Soviet citizens and their international counterparts. The relaxation of press censorship (glasnost’)—alongside policies of restructuring (perestroika) and democratization (demokratizatsiia)—generated frank public discussions of the country’s economic, political, and social problems and an explosion in new grassroots political groups and social movements.^4^ These processes offered fertile ground for grassroots transnational mobilization, as Soviet citizens increasingly looked abroad for the inspiration, information, resources, and moral support required to address deep-seated political, social, and economic problems.

Within this context, HIV-AIDS action offered Soviet citizens new opportunities for transnational exchange and collaboration. This article explores grassroots HIV-AIDS action coordinated in Moscow and Leningrad between 1988 and 1991. HIV and AIDS had been discussed on the pages of the Soviet press from 1985, but 1988 marked a turning point in public perceptions of the epidemic. In late 1988, 270 patients, most of whom were children, were infected with HIV in hospitals across the small town of Elista in the Kalmyk Republic, mainly as a result of contaminated medical equipment.^5^ The Elista tragedy and the many similar incidents that followed shifted public attention away from the prevalence of HIV-AIDS in “risk groups” (namely gay men and sex workers) onto the failures of the Soviet government to protect the health of its citizens.

HIV and AIDS are generally absent from the rich and ever-developing body of scholarship on LGBT experiences in the Soviet Union.^6^ The limited amount of existing scholarship focuses primarily on the overtly homophobic tenor of public discussions of HIV-AIDS in the late 1980s, as well as the inaction of the Soviet government and widespread underreporting of cases.^7^ Literature on Soviet transnational entanglements related to HIV-AIDS has been limited to an exploration of the joint Stasi-KGB misinformation campaign. During Operation Denver, the Soviet and East German security services created and widely disseminated the conspiracy theory that HIV had been created in a lab by US military scientists at Fort Detrick in Maryland.^8^

The Soviet case has also not yet been incorporated into the wider historiography of global AIDS activism, either. First and foremost, the omission is likely a question of timing, as the acceleration of the epidemic in the USSR was overshadowed by the country’s rapid disintegration between 1988 and 1991. Second, the absence of the Soviet Union in scholarship also reflects the specific conditions of Soviet society. Global histories of AIDS activism tend to focus on the instrumental role of LGBT rights groups, who often drew on their existing expertise and connections in their tireless drive for recognition, rights, support, and treatment.^9^ When HIV arrived in the USSR, homosexuality was heavily stigmatized, sex between men was a criminal offense, and there was no publicly visible and well-connected LGBT movement. Public discussions of homosexuality and HIV-AIDS were intensely stigmatizing, which likely explains why there was no activist movement comprised of openly HIV-positive people in the Soviet period.^10^

The Soviet case offers a new perspective on global AIDS action and nuances existing scholarship that draws close connections between AIDS activism and gay activism.^11^ This article advances two principal arguments. First, that because of the specific conditions of late Soviet society, AIDS action was characterized by a dynamic mix of liberal and conversative elements. Hakan Seckinelgin has argued that gay community activism “gave rise to a form of social mobilization generated by particularly disadvantaged groups and their claim to resources on the basis of their citizenship rights”.^12^ In the context of the USSR, certain civil society groups focused on HIV-AIDS widened the frame of disadvantage to include society at large, who they portrayed as at the mercy of a neglectful and uninterested government who showed little concern for public health. Therefore, in the Soviet case, discussions about the destigmatization and liberation of marginalized groups happened concurrently alongside robust critiques of the state healthcare system and the All-Union Soviet government, wherein the focus was squarely on the health of children and the heterosexual majority.

Second, the article argues that Soviet HIV-AIDS action was marked by transnational (and asymmetric) encounters from the outset. The epidemic accelerated at a time when possibilities for international exchange and connection were increasingly possible. Citizen diplomacy initiatives escalated under the conditions of Gorbachev’s reforms, and Soviet citizens entered into dialogue with their international counterparts at citizens’ summits, foreign exchanges, and televised international videoconferences known as space bridges or telemosty.^13^ Just like activists in other state socialist contexts, Soviet citizens who were engaged in HIV-AIDS action could not rely on major institutional or informational support from large segments of society or the state, which quickly pushed their action into a transnational framework.^14^ Soviet citizens sought moral and material support from abroad, and in doing so, bypassed state and party institutions to foster their own connections and challenged the All-Union government’s monopoly on diplomacy and foreign exchange in the field of disease control. HIV-AIDS action was and continues to be strongly influenced by the entanglements between the international political environment and the specific local context.^15^

Therefore, in the Soviet case, the transnational elements of the story tend to privilege major centers of Union-wide mobilization like Moscow and Leningrad, but this does not mean that HIV-AIDS action was not prevalent across other regions of the disintegrating USSR.^16^ Just like elsewhere in West and within the socialist bloc, the Soviet AIDS response was far from monolithic.^17^ Instead, it was characterized by a dynamic engagement between a wide variety of state and non-state actors, the entanglement of liberal and conservative ideas, and a mix of Union-wide and transnational connections.

Examining grassroots HIV-AIDS action means turning away from official collections and assembling an alternative archive from activists’ personal collections, against-the-grain readings of a range of official sources, and oral histories; a practice long familiar to scholars of queer history.^18^ Oral history in particular has long been an essential methodology for the social histories of HIV-AIDS, both for highlighting the social impact of the epidemic and uncovering voices and perspectives that have been marginalized in public discussions.^19^ Turning to alternative sources is essential for studying grassroots HIV-AIDS action in Moscow and Leningrad. State archival collections are shaped by “heteronormative Soviet and Russian information regimes” that tend to silence LGBT perspectives and reflect state homophobia and transphobia, plus Russian state archives are now inaccessible in the wake of Russia’s full-scale invasion of Ukraine.^20^ This article also draws upon oral history interviews, as well as activists’ personal collections and Soviet periodicals that are mostly freely accessible online.^21^ This methodology is not entirely unproblematic, as a source base comprised of publications and reflections by activists is skewed towards their self-representation and does not allow us to fully interrogate the impact and reception of anti-AIDS initiatives. Moreover, this “ad hoc archive” privileges specific voices, namely the perspectives that were published in periodicals that are remotely accessible and the reminiscences of those who are still alive and contactable.^22^ Despite the limitations, this source base provides crucial insight into grassroots AIDS action and allows us to move beyond the viewpoints of state officials and medical professionals, who have tended to dominate the limited existing scholarship on the Soviet case.

HIV-AIDS action in the Soviet Union evolved rapidly across the final years of the country’s existence. This article explores this evolution, first by focusing on attempts to address material shortages in the late 1980s, namely the mobilization of Soviet citizens across republics and regions of the USSR and fundraising efforts targeting individuals and companies based abroad. In these initial appeals for aid, helping the “innocent victims” of HIV-AIDS (such as children) was the primary focus and the impact of HIV-AIDS on “risk groups” (such as gay men, sex workers, and drug users) was omitted. Transnational material support rapidly evolved into face-to-face interactions between Soviet activists and their international counterparts. The second part of the article explores the coordination of US-Soviet action in Moscow and Leningrad around the interconnected issues of gay rights and HIV-AIDS. Examining this evolution from material support to face-to-face interactions shows how grassroots action was marked by a mix of conservative and liberal elements, as well as the centrality of transnational connections to both currents of activism.

## Bypassing the state: Ogonek anti-AIDS and international fundraising

The transnational nature of grassroots Soviet HIV-AIDS action arose from equipment shortages within the USSR. Despite profound innovation in healthcare and medical sciences and investment in experimental medical research, the Soviet government consistently underfunded medical services and healthcare infrastructure could not meet the needs of the population. The Soviet healthcare system was plagued by a poor supply of medications and equipment, deteriorating and outdated facilities, and low morale amongst medical workers.^23^ Despite attempts to revitalize the Soviet economy in the late 1980s, there was a chasm between production targets and the actual number of available products that were essential for preventing the spread of HIV, such as latex condoms, surgical rubber gloves, and disposable syringes. For example, the state ministry in charge of manufacturing surgical gloves had produced less than a third of the required amount in 1988.^24^ By this point, the Soviet government still had not established a domestic facility to produce syringes and continually failed to meet the production targets for needles, spectrophotometers, and sterilization devices. When challenged by journalists, government representatives tended to shirk responsibility and point the finger at individual doctors and production workers, thus reverting to the “familiar Soviet discourse of blame” that prioritized individual moral failings over systemic problems.^25^

In the Gorbachev-era USSR, as cases of HIV and AIDS slowly crept up and the government failed to take responsibility for the situation, grassroots organizations looked abroad for the essential resources required to stem the tide of the epidemic. In 1989, employees of the mass-circulation magazine Ogonek (Spark) opened a dedicated charitable fund named Ogonek anti-AIDS (Ogonek-antiSPID). The founders of the Ogonek anti-AIDS fund were adamant that their organization was entirely non-state affiliated and its early patrons were a who’s who of the country’s “radical intellectual elite.”^26^ Published in Moscow, Ogonek was a weekly illustrated magazine that had a circulation of several million by the late 1980s.^27^ Following the installation of Vitaly Korotich as editor in 1986, the magazine became a formidable bastion of social critique and a mobilizing force for the glasnost’-era intelligentsia.^28^ The Ogonek anti-AIDS fund was established immediately after the mass infection of infants during the Elista tragedy of late 1988. Ogonek extensively covered the disaster, publishing both articles and reactions from the public in the form of readers’ letters.^29^

Appeals for donations to purchase disposable syringes, blood transfusion systems, intravenous catheters, and other types of medical equipment were regularly published in Ogonek throughout 1989–1991. Although Soviet citizens could donate to the fund in rubles, donations in so-called ‘hard currency’ (valiuta, meaning stable foreign currency) were preferred because equipment had to be purchased from abroad. The ruble was non-convertible and therefore excluded from global financial systems.^30^ In bypassing the state and appealing to the public, staff at Ogonek drew on the longstanding practice of turning to informal networks to address social problems. Soviet citizens largely relied on the broader “economy of favors” to address the deficiencies of the command economy and deal with the constant shortages characteristic of life in the Soviet Union.^31^

Ogonek’s wide circulation ensured that their call for hard currency reached a broad audience, and donations of goods and money quickly began to pour in from across the USSR. The Institute of Nuclear Physics in the Siberian town of Akademgorodok donated an ILU-6 electron accelerator, which could be used to sterilize 50,000 disposable syringes per hour and which would cost at least $1 million to import into the Soviet Union.^32^ Cash donations were also received from the crew of the Soviet Black Sea cruise-ship “Shota Rustaveli,” staff at Moscow’s Red October confectionary factory, the Soviet Red Cross, and the Russian Christian Democratic Movement, an anti-communist and pro-market economy political coalition that was founded in spring 1990.^33^ Contestants of the 1989 Miss Photo USSR competition agreed that the prizes (which included a number of fur coats and a brand new Zhiguli car) ought to be auctioned off abroad in order to transfer more hard currency to the Ogonek anti-AIDS fund. Winning the competition meant working as a model for foreign advertising agencies and magazines, and all finalists agreed to donate 10 per cent of their salary to the fund if they emerged victorious.^34^ Ogonek’s fundraising efforts were supported by numerous Soviet citizens from various segments of society, but they were not endorsed by the Kremlin. Gorbachev did not engage with Ogonek anti-AIDS, nor did he respond to the appeals that they directly addressed to him.^35^

Ogonek managed to generate support from the Soviet population and international groups because they shifted the narrative on AIDS away from “at risk” groups to a more socially conservative framing focused upon the infection of children and hospital patients due to the Soviet government’s underfunding of healthcare services. In March 1990, the magazine published an appeal to the government and Supreme Soviet of the USSR (the country’s principal legislative body), which included a list of demands to stem the tide of the country’s HIV epidemic. The authors of the appeal questioned what kind of country the USSR was if citizens could not trust state institutions and the healthcare system: “Unlike civilized countries of the world, where the main source of infection is drug addicts, homosexuals and prostitutes, in our country medical institutions are hotbeds of the disease. Think about it: almost all children with the disease became infected in hospitals!”^36^ Here, the authors recycled well-worn stereotypes about the decadent and debauched West that were common in Soviet discourse on sex and sexuality.^37^ The authors of the appeal looked to the United States for an example of a government taking the epidemic seriously and reminded the Soviet government that AIDS spending in the US was set to exceed military spending in 1990.

As well as addressing Soviet citizens, Ogonek appealed to its readers located outside the USSR. Since the relaxation of isolationist foreign policy following the death of Stalin, the Soviet government endeavored to choreograph interactions between Soviet citizens and foreigners both to prevent ideological contamination and reputational damage to the USSR.^38^ Ogonek’s appeals bypassed the state entirely and requested foreign aid to supplement the state’s inadequate healthcare services and the deficiencies of the planned economy. Here, the magazine occupied a precarious position, acting almost as an NGO and vocal critic of the party-state, but all while remaining dependent on state resources in order to function.^39^ In one 1989 appeal, Ogonek directly addressed the Soviet diaspora and their wider international audience:

We call on all developed countries of the world to help us. We turn to charitable (religious and secular) organizations, foreign companies, wealthy people, our compatriots now living abroad, and ask you to transfer hard currency to the anti-AIDS account. We turn to enterprises—those who manufacture disposable medical products—and ask you to sell to us on friendly terms. We also turn to organizations abroad that could sponsor charitable exhibitions, sports matches, the sale of painting by modern Soviet artists, and concerts by Soviet and foreign “stars” to help the anti-AIDS account.^40^

In their international appeals, Ogonek staff cultivated an image of a helpless population who were at the mercy of their negligent government and therefore required foreign intervention. “We ask for salvation. We are unable to protect ourselves from AIDS, so protect us!” one emotional Ogonek appeal read, before reminding readers abroad that “your children are not in danger of becoming infected from a simple infection, from a dirty syringe. Save our children too.”^41^ When expressing their very real concerns about the chronic underfunding of Soviet healthcare services, the initiators of the Ogonek anti-AIDS fund drew on well-established frameworks of Soviet inferiority that would have been familiar to Western audiences. For example, US American journalists reporting from the Soviet Union throughout the 1960s-1980s fixated on the material hardships of Soviet life and presented Soviet citizens as passive victims of their government’s false promises.^42^ In asking for “salvation,” Ogonek’s appeals for donations tapped into longstanding Cold War stereotypes about Soviet citizens as godless communists who required spiritual liberation from believers on the other side of the iron curtain.^43^

Ogonek also provided a platform for groups to publish their own targeted requests for foreign aid. In autumn 1989, a group of Russian Orthodox priests used Ogonek to appeal to international religious organizations, particularly the Russian Orthodox Church abroad, for spiritual and material support. The group asked for an International Day of Prayer and instructed donors to entirely bypass the Soviet government, which was “not able to feed and clothe its citizens, nor protect them from AIDS”; something that the priests absolutely could not condone.^44^ “We ask for help not for our state,” they wrote, “we ask you to help us save our children, both the faithful and prodigal” (bludnye). The Ogonek anti-AIDS fund provided Soviet citizens with a channel for forging transnational connections and offered opportunities to challenge the government’s monopoly on diplomacy and the exchange of foreign goods.

Individuals and organizations from around the world answered Ogonek’s appeals for aid. For example, Bob Guiccione and Kathy Keaton—the owners of the US American media conglomerate General Media Incorporated—sent a large financial donation and shipped 85,000 disposable syringes to the Soviet Union, which were delivered to a children’s hospital in Nukus, the capital of the Karakalpak Autonomous Soviet Socialist Republic, which was in the territory of the Uzbek Republic.^45^ Donations from other foreign companies were sent to children’s hospitals in the port cities of Vladivostok, Batumi, and Odesa.^46^ The Soviet-Korean Cooperation Sokora sent 32,800 disposable syringes from Seoul and the Bulgarian firm KAM’s donation of 10,000 syringes was sent to a maternity hospital located in a small city 85km north of Moscow.^47^ A gift of 15,000 disposable syringes was sent by the Soviet-Indian charity Panchsheel-Perestroika, after which it was delivered to the city hospital in Shakhty in southwestern Russia.^48^ In Japan, the newspaper Sekai Nippo and the NGO “Global Rainbow Ship” organized a shipment of 38,100 disposable syringes to the Soviet Union.^49^

Ogonek’s appeal also opened avenues for collaboration between Soviet citizens and the Soviet diaspora. A group of Soviet women living in Alexandria, Egypt, driven by “the pain in [their] hearts for all the numerous problems in our Motherland”, donated 2880 disposable syringes and requested that they be sent directly to the children’s department of the Burdenko Neurosurgery Institute in Moscow.^50^ Cellist and Soviet emigree Boris Pergamenshchikov organized a charity concert at an Evangelical Church in Bergisch Gladbach, Germany, and transferred 2500 Deutsche Marks to the Ogonek fund.^51^ Exiled Soviet dissidents also played a major role in fundraising for Ogonek anti-AIDS. Poet and art collector Aleksandr Glezer emigrated from the USSR in 1974 when an open-air exhibition of unofficial art that he had organized was destroyed by the Soviet authorities.^52^ Glezer organized a series of international charity exhibitions at his Museum of Contemporary Russian Art in Exile in Paris.^53^ The exhibition included works donated by Russian emigree artists, which were subsequently sold and 25,000 francs was transferred to the Ogonek anti-AIDS fund.^54^

With the inaccessibly of the archival collections related to Ogonek’s editorial office, we do not have a way to verify whether their fundraising campaign had a tangible material impact upon people living with HIV and AIDS in the USSR. Even still, Ogonek’s campaign offers insight into the conceptualization of AIDS as an urgent social problem amongst Moscow intellectuals and the discursive strategies that they employed to present the threat of AIDS in a manner that was socially acceptable. For example, in framing its appeals around protecting children from HIV and AIDS, Ogonek*’*s fundraising activities were part of broader transnational currents of AIDS action that shifted attention away from “at risk” groups towards the so-called “innocent victims” of the epidemic to generate support. For instance, in the USA, the move away from discussing drug users, sex workers, or gay men to focusing on the protection of children and the prevention of infection through inadequate medical equipment made it possible for moral conservatives to join the struggle against AIDS.^55^ Politicians and journalists in various international contexts have used the infection of children and mothers to illustrate the dangers of HIV and AIDS within the so-called “general population,” an evasive term used to refer to the heterosexual majority and a category that tends to exclude intravenous drug users, sex workers, and gay men.^56^ The discursive construction of the category of blameless child victim of AIDS implied that the infection of other groups who were deemed to be less innocent (such as sex workers and intravenous drug users) were not worthy of sympathy, attention, or support.^57^ Jessica Ogden and Laura Nyblade have explored the ways in which HIV and AIDS-related stigma interacts with preexisting stigmas in discussions of HIV infection. The coalescence of multiple forms of stigma has produced an “Innocence-to-Guilt” continuum where HIV-positive children and sex workers/drug users are placed at opposing ends.^58^ In the Soviet Union, Ogonek’s focus on protecting children was particularly rousing given the public uproar about the mass infection of children in hospitals in Elista, Volgograd, Stavropol, and Rostov-on-don throughout 1989 and 1990, as well as the fact that children were disproportionately overrepresented in publicly-available data on HIV infections in the late 1980s and 1990s.^59^ The intense stigmatization, direct/indirect criminalization, and at times denial of the existence of sex workers, gay men, and drug users also made it very unlikely for HIV-AIDS action to be centered around these groups in the Soviet Union.^60^

## The Americans are coming: Foreign delegations and Soviet HIV-AIDS action

Soviet HIV-AIDS action quickly evolved from international fundraising to face-to-face interactions between Soviet activists and their international counterparts. In October 1990, a team of HIV-AIDS educators from the USA arrived in Moscow. The delegation had been assembled by family and relationship therapist Stuart Altschuler, and included Mitchell Kushner, a HIV-practicing physician, and Wendy Arnold, a specialist in the development of HIV-prevention programs for young people, as well numerous HIV-positive activists. The delegation was part of the dramatic surge in US-Soviet citizen diplomacy that occurred during the mid-1980s following the expansion of exchange and travel initiatives by Ronald Reagan and Mikhail Gorbachev.^61^ Altschuler attended the second Citizens’ Summit in Moscow in January 1990, which was an international conference co-sponsored by the Soviet-American Centre for Dialogue and Soviet Peace Committee. The event attracted hundreds of delegates and acted as a springboard for the establishment of hundreds of joint ventures between Soviet and US citizens.^62^ Following the Citizens’ Summit, Altschuler co-founded the International Centre for Better Health (ICBH): a Soviet-American non-profit focused broadly on women’s and children’s health care, as well as HIV education and prevention.^63^ Throughout the 1990s, Altschuler worked with the American and Soviet members of the ICBH to bring US delegations of educators, administrators, and HIV-positive activists to the Soviet Union, and, later, the Russian Federation.

Members of US delegations regarded their visits to the Soviet Union as purposeful for many reasons. First, they provided an opportunity to share resources and information with the hope of lessening the epidemic and improving the plight of people living with HIV and AIDS in the Soviet Union.^64^ An article covering the October 1990 delegation’s visit included interviews with Marjorie Mason, who was HIV-positive, and Michael Reynolds, who had been diagnosed with AIDS back in 1987 and who was the director of the West Hollywood Centre for Gays and Lesbians. The publication of interviews with HIV-positive individuals was hugely significant as no HIV-positive Soviet citizens had publicly declared their status in the media at this point. Both Reynolds and Mason passionately advocated for the rights of HIV-positive people and emphasized the importance of working with high-risk groups (specifically drug users, gay men, and sex workers) by promoting safe sex and distributing disposable syringes and condoms.^65^ US visitors were also keen to counter misinformation about HIV and AIDS. Stuart Altschuler deliberately drank from the same water glass as Michael Reynolds during an event held at a Moscow medical school to the sound of a cacophony of gasps from the audience.^66^ US visitors also provided their hosts with a communication channel for the circulation of accurate information about the Soviet epidemic outside the borders of the USSR, which served to remedy the misinformation spouted by the Soviet government.

Soviet commentators were keen to mine the experiences of their US counterparts to prevent the spread of infection and improve the lives of people living with HIV and AIDS. In 1991, SPID-info*—*a well-known glasnost’-era magazine dedicated to discussing issues related to sex—published an interview with Sergei Trofimov, who founded the first “Anti-AIDS” anonymous 24-hotline in the USSR.^67^ Trofimov and other Soviet psychotherapists set up the hotline after meeting with US HIV and AIDS specialists who visited the Soviet Union and warned the Soviets to act quickly and not repeat the mistakes that they had made during the early stages of the epidemic in the United States. In the article, Trofimov discussed Michael Reynolds at length, using his case to stress the importance of not ostracizing people with HIV and AIDS and the urgent need for infected individuals to stay as mentally and physically active as possible. “Reynolds can serve as an example for how to deal with the infection,” Trofimov wrote, before explaining how the young man continued to participate in AIDS activism and even trained as a bodybuilder following his diagnosis, activities which he claimed helped his body to fight off the infection.^68^ The SPID-info editors tacked a takeaway point to the end of the article informing readers that “friendship cannot cause AIDS” and encouraging them to care for and support people with HIV and AIDS who were likely undergoing severe psychological stress. Given that the Soviet Union’s epidemic was in its early stages in 1990–91, with cases not yet in the thousands, the experiences of US activists had almost a prophetic quality and were deemed important pieces of evidence for dictating the course of events in the USSR.^69^

In 1991, the International Gay and Lesbian Symposium and Film Festival was convened in Leningrad and Moscow. The event was organized by the International Gay and Lesbian Human Rights Commission (IGLHRC), which was founded in summer 1990 by US activists Julie Dorf and Jim Toevs, and Roman Kalinin, a prominent Soviet gay activist and editor of the Soviet Union’s first queer magazine Tema.^70^ The symposium itself was another example of US-Soviet grassroots citizen diplomacy. When making the arrangements for their sixty-nine foreign guests, the organizing committees in Leningrad and Moscow decided to bypass the Soviet government entirely and arrange all the visas, transport, accommodation, catering, and interpreters themselves.^71^ The ten-day event included a packed schedule of plenums and workshops, as well as visits to an AIDS treatment center and to a prison to meet with men who had been convicted under the Soviet Union’s anti-sodomy criminal article. The accompanying film festival was sponsored by the San Fransisco non-profit Frameline, the organizers of the oldest continuous LGBT film festival in the world.^72^ During the Moscow leg of the trip, Symposium participants engaged in direct action, including the USSR’s first gay rights demonstration on the steps of the Bolshoi Theatre and a condom distribution demonstration and kiss-in outside the Moscow City Soviet.^73^

The International Symposium contributed to the broadening of Soviet public discussions about HIV-AIDS and helped to increase the visibility of people living with HIV. Plenary sessions on the AIDS epidemic were held at both the Leningrad and Moscow legs of the symposium, during which Soviet and US activists and experts engaged in “heated discussions” with members of the audience about the criminalization of HIV transmission and lack of anonymity during HIV testing in the Soviet Union.^74^ The presence of dozens of Western activists at demonstrations for gay rights and AIDS awareness ensured that the events were widely reported in the Soviet press and on television.^75^ In the opinion of US attendee Tom Boellstorff, the fact that the demonstrations were “part of a larger event that had a lot of eyes” likely provided Soviet participants with a layer of protection from the police.^76^ The International Symposium also pushed public discussions of HIV and AIDS in the Soviet Union beyond abstract medicalized generalizations. As noted in the article’s introduction, Gennady Roshchupkin spontaneously became the first Soviet citizen to publicly disclose their HIV status during one of the Symposium sessions in Leningrad. In autumn 1991, Roshchupkin appeared in the Soviet press following the six-day hunger strike that he undertook while being treated at the only AIDS treatment facility in the Soviet Union—the Sokolinaya gora AIDS clinic in Moscow. The purpose of the hunger strike was to demand the establishment of a fully-funded state AIDS programme, the repeal of the anti-sodomy criminal article, and the improvement of the dismal treatment facilities available to HIV-positive people.^77^ After this, Roshchupkin gave a number of interviews to the press and on the radio, and even appeared on an episode of Vlad Listyev’s TV talk show “Tema” dedicated to discussing AIDS in December 1992.^78^

For the IGLHRC, the Symposium was an opportunity to provide material and technical support to Soviet activists in the form of equipment and digital skills. When arriving in the Soviet Union that summer, Julie Dorf carried a canvas bag of 10,000 condoms through Soviet customs, much to the bemusement of the customs officer on duty.^79^ Along with the sixty-nine US delegates, the IGLHRC shipped over several Macintosh computers, printers, and photocopy machines to help gay and lesbian groups publish without the interference of the Soviet government. After the Symposium, Tom Boellstorff stayed in Moscow for a few months in an apartment paid for by the IGLHRC to teach Soviet activists how to use the equipment and navigate the English-language systems of both the Macintoshes and publishing software QuarkXPress.^80^ This equipment was used to print the gay and lesbian magazine Ty (You), which regularly published articles on HIV and AIDS and donated a portion of its profits to a charity that supported HIV-positive people My i Vy (Us and You), which was established by Roshchupkin in 1991.^81^ The equipment and skills provided by the IGLHRC also had a broader significance beyond discussions of HIV and AIDS. During the August 1991 coup when communist hardliners attempted to remove Gorbachev from power, the computer and printer at Tom Boellstorff’s apartment were used to retype and photocopy statements by Boris Yeltsin and other pro-democracy leaders, some of which even carried the logo of the gay magazine Tema at the insistence of Roman Kalinin.^82^

The International Symposium of the summer of 1991 was not the first initiative to connect gay liberation with the issue of AIDS, nor was it the only transnational LGBT event held within the USSR.^83^ That being said, the Symposium was an important citizen diplomacy initiative that resulted in tangible knowledge, skills, and cultural exchange between US Americans and Soviets. Co-founder of IGLHRC Julie Dorf reflected on the significance of this kind of international community building in the pre-internet era, when finding one another was like a “hidden gem”, when interactions were based upon “generosity and understanding”, and where connections often resulted in “lifelong friendships and changes that were very eye-opening in both directions.”^84^ Other Symposium attendees reflected on the feelings of connection and similarities between US and Soviet delegates, both because of a shared identity and their lack of organizational experience. Tom Boellstorff noted at the time of the Symposium, that IGLHRC had “zero money and no paid staff,” had not yet been officially registered as a non-profit, and was run out of an activist’s apartment in San Fransisco’s Mission District.^85^ Julie Dorf also explained that IGLHRC began “in [her] kitchen with a ragtag group of immigrants and activists” who had very little in the way of resources and experience.^86^ Although some Symposium attendees were independently wealthy, Boellstorff described the majority of North Americans who visited Leningrad and Moscow in summer 1991 as “broke gay and lesbian folks.” North American visitors to the USSR tended to acknowledge the material inequalities between the two groups—unavoidable as the North Americans were bringing technology and medical supplies—but stressed their commonalities with the Soviets in terms of resources and organizational experience.^87^ Similarities were also noted by Gennady Roshchupkin, who claimed that “they [US activists who did not work for large NGOs] had no experience, and we had no experience. We were building something completely new together.”^88^

Although US-Soviet collaborations fostered long-lasting solidarities, this kind of transnational mobilization had its own hierarchies that were infused with the geopolitics of inequality. While bilingual US Symposium organizers Julie Dorf and Masha Gessen endeavored to avoid “even the appearance of importing western culture” and used Russian-language terminology throughout the event (for example, opting for the terms “*goloboi*” (light blue) and “*rozovoi*” (pink) in place of “gay” or “lesbian”), they were met with a “hungry invitation” for Western culture and resources.^89^ Soviet attendees’ “hunger” for information is reflective of the broader (and much critiqued) hierarchies that continue to undergird LGBT activism, within which “the flow of cultural knowledge and practice seems to be one way: from the West to the rest.”^90^ The asymmetrical relationship between US and Soviet Symposium attendees went deeper than access to information. Roshchupkin explained that equality was not possible between the two groups because “equality is between more or less the same people, but we were not the same, and therefore, in principle, we were unequal. We were from different worlds, with a different reality, different social protection, and very different knowledge about the reality in which we were all in. The understanding—both social and medical—was also very different.” Julie Dorf noted that US activists “felt very much like outsiders and marginalized” in the context of the Reagen era and could relate to their Soviet counterparts “in terms of the closet and [issues of] safety”.^91^ However, there was an “inherent inequality and power dynamic” tipped in favor of US activists who had access to material resources and the freedom to travel because of their “blue passports”, unlike the vast majority of Soviet activists. While US-Soviet transnational solidarities borne of HIV-AIDS action were deeply meaningful for both groups, they were unavoidably asymmetric and inflected by broader global material and epistemological inequalities. This asymmetry would become ever more apparent as LGBT activism became increasingly “NGOized” after the Soviet collapse and local LGBT rights initiatives in the postsocialist world became ever more reliant on Western funding in order to function.^92^

## Conclusion

In the USSR’s twilight years, transnational HIV-AIDS activism provided a welcome corrective to the inaction of the All-Union Soviet government. In an atmosphere of the intense stigmatization of people living with HIV and AIDS, Soviet citizens wanting to address the epidemic could not rely on the institutional support of the state nor the financial support of broad segments of the population, and instead looked abroad for material and moral sustenance. The transnational connections that emerged within Moscow and Leningrad during the Gorbachev-era Soviet Union would outlast the country’s disintegration. Ogonek anti-AIDS continued to fundraise and produce informational materials within the Russian Federation throughout the 1990s. After International Gay and Lesbian Symposium of summer 1991, IGLHRC lobbied US pharmaceutical manufacturers to donate medication for individuals with HIV and AIDS to the Soviet Union and helped to coordinate pen-pal projects that connected people living with AIDS in the US and USSR, as well as a US-USSR gay deaf pen pal project. North American activists who visited the Soviet Union in 1990/1991—including several Symposium attendees—frequently visited the Russian Federation and continued to collaborate with their Russian counterparts throughout the decade that followed. These transnational connections persisted across political and economic collapse, but their existence was largely made possible because of the specific context of late Soviet society where increased public awareness and anger following the Elista tragedy, widespread civil mobilization, and the explosion of citizen diplomacy initiatives encouraged Soviet citizens to look beyond the country’s borders for solidarity and solutions.

Soviet HIV-AIDS action was a dynamic interaction that was characterized by the entanglement of liberal and conservative ideas. In focusing on the impact of HIV-AIDS on “innocent” groups (like infants infected within medical settings) Ogonek participated in the transnational flow of morally conservative ideas and deliberately sidelined the impact of the epidemic on groups deemed to be “at risk”, such as gay men, sex workers, and drug users. As HIV-AIDS action developed into the 1990s, new currents emerged that focused specifically on gay rights and liberation. Soviet activists fostered connections with their counterparts in the United States to share resources, learn from one another, and build transnational solidarities. These transnational solidarities were deeply meaningful to both parties, but were underpinned by inequalities in terms of knowledge, economic resources, and passport access.

## Figures and Tables

**Figure 1:**
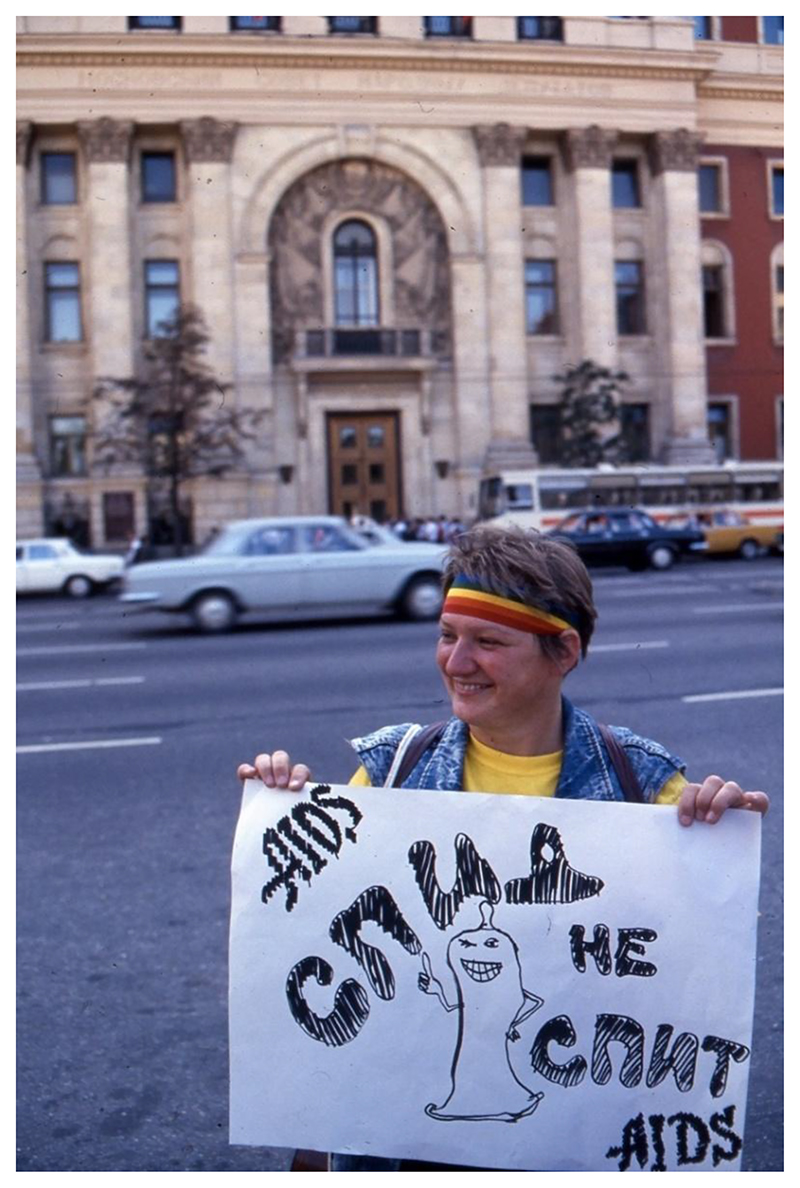
Demonstration outside the Moscow City Soviet, 30 July 1991. The sign reads “AIDS does not sleep.” Source: Personal collection of Tom Boellstorff.

**Figure 2:**
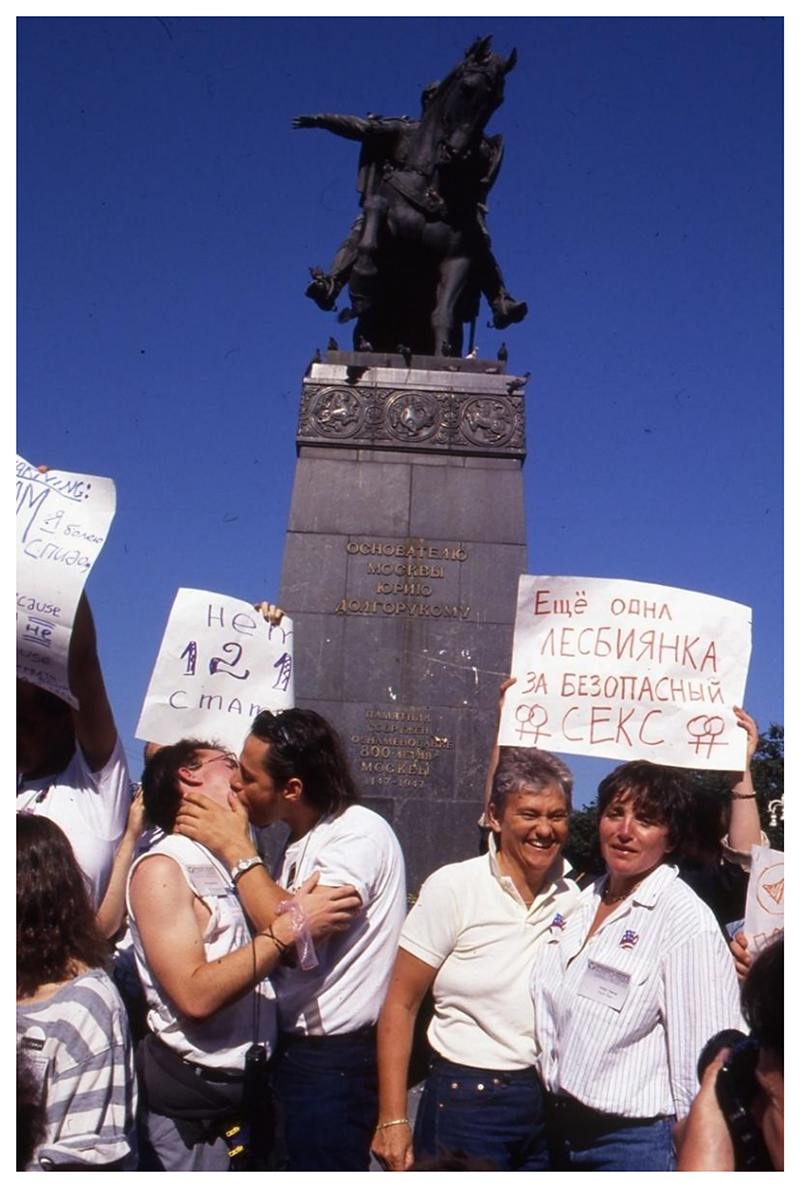
Demonstration outside the Moscow City Soviet, 30 July 1991. The signs read “No Article 121” (the anti-sodomy article in the Soviet criminal code) and “Another lesbian for safe sex”. Here, US activist Tom Boellstorff (left) can be seen kissing another symposium attendee. Source: Personal collection of Tom Boellstorff.

**Figure 3:**
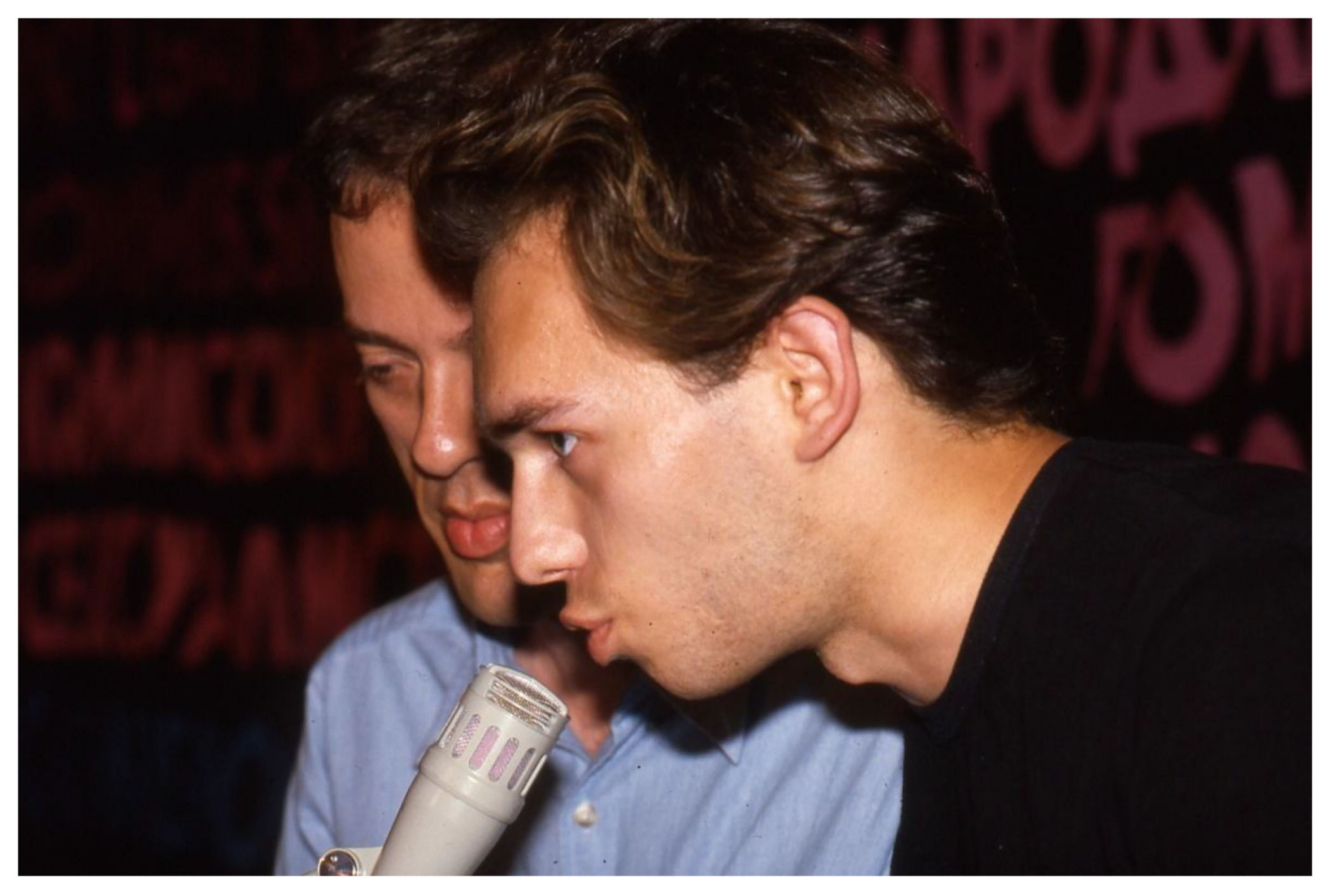
Gennady Roshchupkin speaking at the International Gay and Lesbian Symposium and Film Festival, July 1991. Source: Personal collection of Tom Boellstorff.

**Figure 4:**
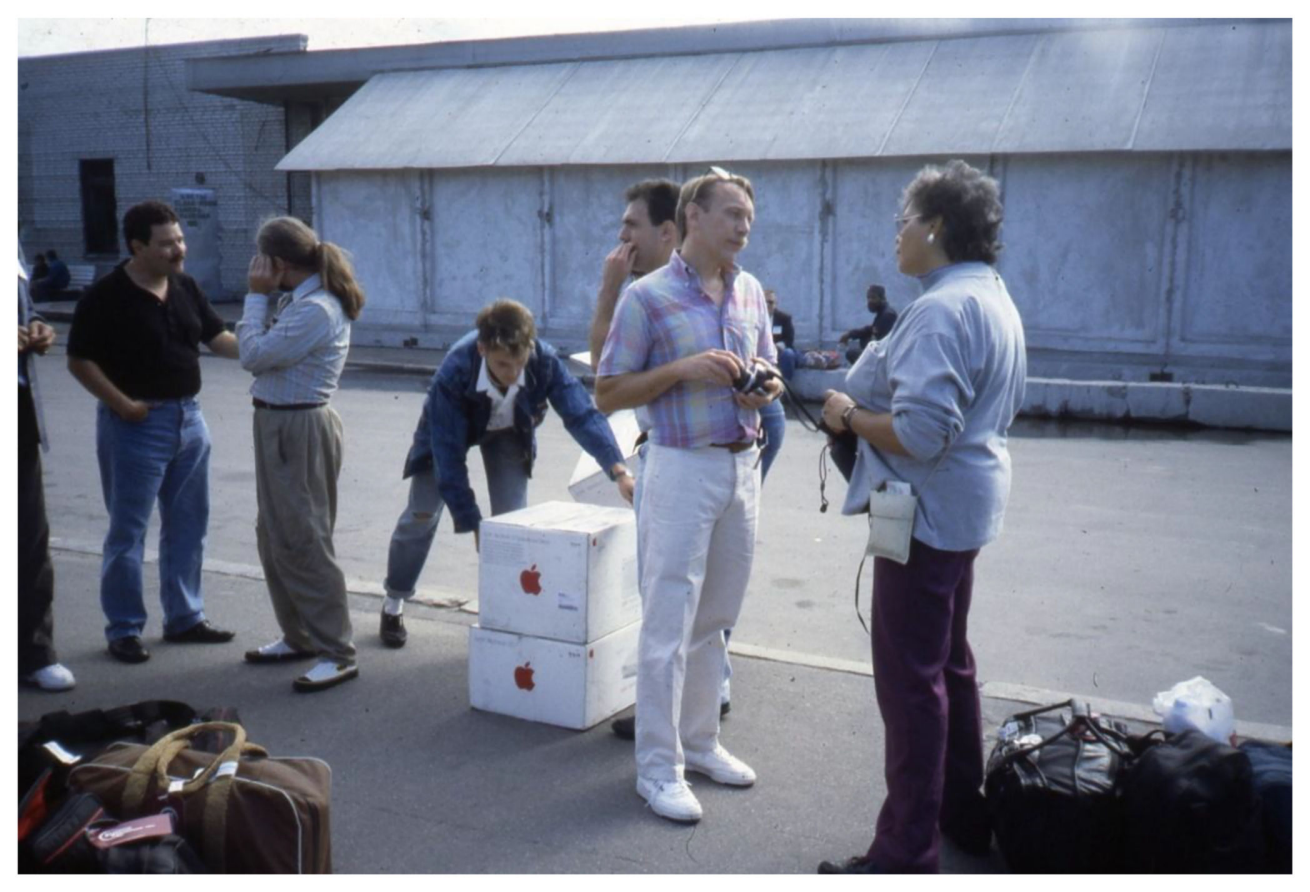
The Macintosh computers from IGLHRC being unloaded during the International Symposium. Source: Personal collection of Tom Boellstorff.

**Figure 5:**
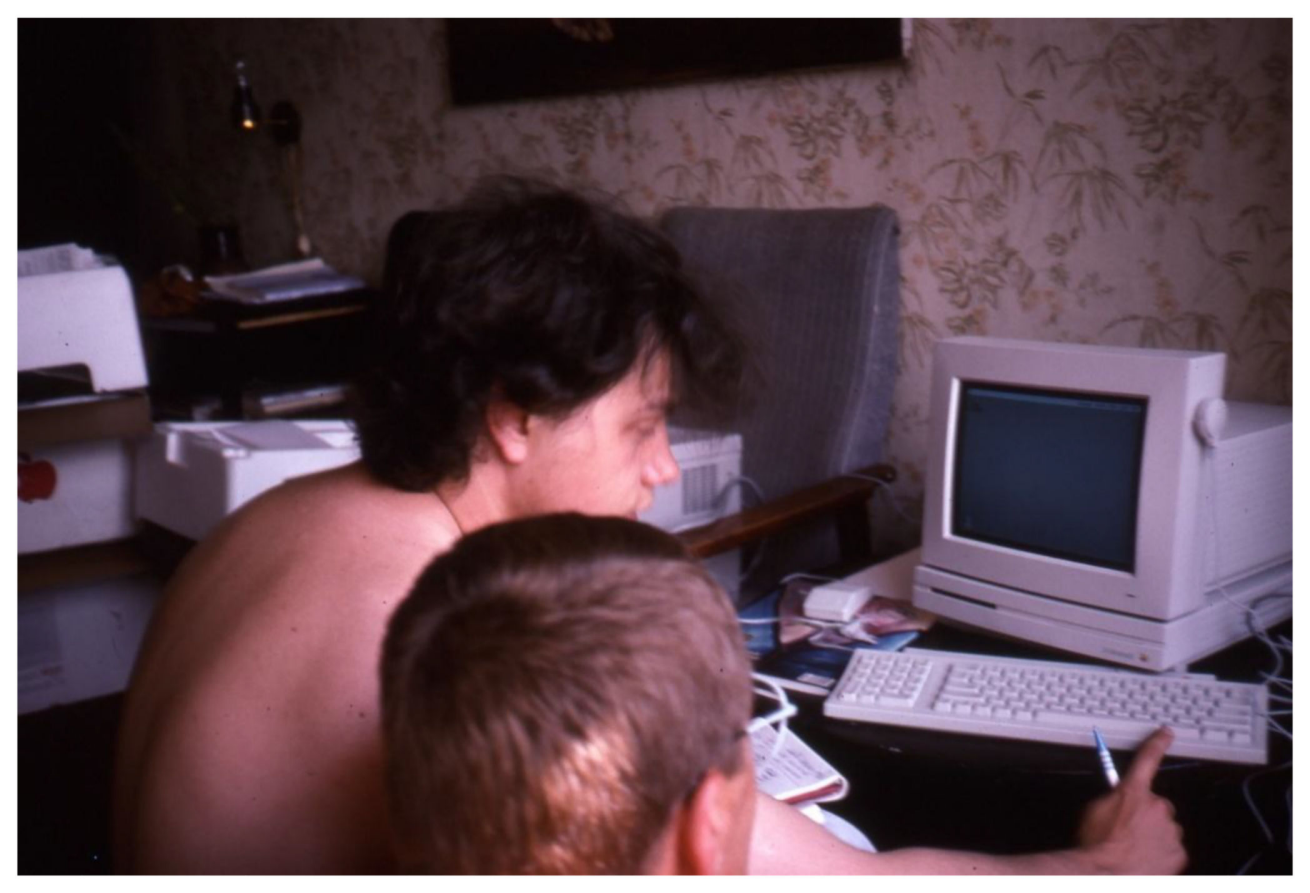
One computer in use at Tom Boellstorff’s apartment near Akademicheskaya metro station, Moscow, August 1991. Source: Personal collection of Tom Boellstorff.

## Footnotes

1 List of symposium attendees from the personal collection of Tom Boellstorff.

2 Interview with Gennady Roshchupkin conducted on Zoom, 29 March 2023. All interviews conducted for this article were based on the principles of confidentiality and informed consent, and I have ensured that the interviewees are happy for their names to be used throughout.

3 Gennadii Roshchupkin, “Aktivizm – eto neravnodushie”, interview published on Life4me+ https://life4me.plus/ru/blog/aktivizm-6191/ (accessed 30 March 2023). Roshchupkin is referred to as Russia’s first AIDS activist in David Tuller, Cracks in the Iron Closet: Travels in Gay and Lesbian Russia (Boston: Faber & Faber, 1996), 210.

4 Judith B. Sedaitis and Jim Butterfield ed. Perestroika From Below: Social Movements In The Soviet Union (London: Routledge, 1991); Vladimir Brovkin, “Revolution from Below: Informal Political Associations in Russia, 1988-1989,” Soviet Studies 42, no. 2 (1990): 233–57.

5 Ekaterina Serova et al. “The 1988 Elista Outbreak and the Epidemiology of the HIV subtype G in Russia,” Virus Evolution 4, no. 1 (2018): 54–55.

6 Dan Healey and Francesca Stella ed. “Dissidences sexuelles et de genre en URSS et dans l’espace postsoviétique,” Cahiers du monde russe 62, no. 2-3 (2021); Rustam Alexander, Regulating Homosexuality in Soviet Russia, 1956–91: A Different History (Manchester: Manchester University Press, 2021); Dan Healey, Russian Homophobia from Stalin to Sochi (London: Bloomsbury, 2017); Arthur Clech, “Between the Labor Camp and the Clinic: Tema or the Shared Forms of Late Soviet Homosexual Subjectivities” Slavic Review 77, no. 1 (2018): 6–29.

7 Rustam Alexander, “AIDS/HIV and Homophobia in the USSR, 1983-90,” Kritika 24, no. 1 (2023): 121–50; Rustam Alexander, Red Closet: The Hidden History of Gay Oppression in the USSR (Manchester: Manchester University Press, 2023) 197–239; Kārlis Vērdiņš and Jānis Ozoliņš, “The Latvian LGBT Movement and Narratives of Normalization,” in LGBTQ+ Activism in Central and Eastern Europe Resistance, Representation and Identity, ed. Radzhana Buyantueva and Maryna Shevtsova (Cham: Palgrave, 2020), 242–45; Murray Feshbach, “The Early Days of the HIV/AIDS Epidemic in the Former Soviet Union” in HIV/AIDS in Russia and Eurasia, ed. Judyth L. Twigg (New York: Palgrave Macmillan, 2006), 7-32; Ulla Pape, The Politics of HIV/AIDS in Russia (London: Routledge, 2014).

8 Douglas Selvage, “Operation ‘Denver’: The East German Ministry of State Security and the KGB’s AIDS Disinformation Campaign, 1985–1986 (Part 1),” Journal of Cold War Studies 21, no. 4 (2019): 71–123; Douglas Selvage, “Operation ‘Denver’: The East German Ministry for State Security and the KGB’s AIDS Disinformation Campaign, 1986–1989 (Part 2),” Journal of Cold War Studies 23, no. 2 (2021): 4–80.

9 Jeremy Youde, “The Global HIV/AIDS and LGBT Movements” in The Oxford Handbook of Global LGBT and Sexual Diversity Politics, ed. Michael J. Bosia et al (Oxford: Oxford University Press, 2019), 301–13; Christophe Broqua, Action = Vie: A History of AIDS Activism and Gay Politics in France (Philadelphia: Temple University Press, 2020).

10 On this intense stigmatization, see Siobhán Hearne, “AIDS and the End of the Soviet Union,” Past & Present (2025) and Alexander, “AIDS/HIV and Homophobia.”

11 Broqua, Action = Vie; Jennifer Power, Movement, Knowledge, Emotion: Gay Activism and HIV/AIDS in Australia (Canberra: Australian National University Press, 2011); Deborah B. Gould, Moving Politics: Emotion and ACT UP’s Fight Against AIDS (Chicago: University of Chicago Press, 2009).

12 Hakan Seckinelgin, The Politics of Global AIDS: Institutionalization of Solidarity, Exclusion of Context (Cham: Springer, 2017), 64.

13 Much of the literature focuses on US-Soviet citizen diplomacy: David Scott Foglesong, “When the Russians Really Were Coming: Citizen Diplomacy and the End of Cold War Enmity in America,” Cold War History 20, no. 4 (2020): 419–40; Helene Keyssar, “Space Bridges: The US-Soviet Space Bridge Resource Centre,” PS: Political Science & Politics 27, no. 2 (1994): 247–53; Steve Brigham, “The American-Soviet Walks: Large-Scale Diplomacy at Glasnost’s Outset,” Peace & Change 35, no. 4 (2010): 594–625; Matthias Neumann, “Children Diplomacy During the Cold War: Samantha Smith’s Visit to the ‘Evil Empire,’” History 104, no. 360 (2019): 275–308.

14 Łukasz Szulc, Transnational Homosexuals in Communist Poland: Cross-Border Flows in Gay and Lesbian Magazines (Cham: Palgrave, 2017), 116. In this special issue, Maja Lukanc shows how Slovenian activists tapped into transnational resources due to an domestic information deficit and their ability to read in multiple languages.

15 Hakan Seckinelgin, International Politics of HIV/AIDS: Global Disease-Local Pain (London: Routledge, 2008).

16 For example, HIV-AIDS action in Riga in the same period was connected with the broader Latvian independence movement, Hearne, “AIDS and the End of the Soviet Union.”

17 Joanna Folland, “‘Not Even the Highest Wall Can Stop AIDS’: Expertise and Viral Politics at the German-German Border,” Central European History (2023): 1–15.

18 For a rich discussion of the opportunities and challenges of queer archives, see Anjali Arondekar, Ann Cvetkovich, Christina B. Hanhardt, Regina Kunzel, Tavia Nyong’o, Juana María Rodríguez, and Susan Stryker, “Queering Archives: A Roundtable Discussion” Radical History Review 122 (2015): 211–31. On the

19 The literature on oral histories of HIV and AIDS is vast, so a few select examples: Agata Dziuban, Eugen Januschke, Ulrike Klöppel, Todd Sekuler and Justyna Struzik, “The European HIV/AIDS Archive: Building a Queer Counter-Memory” in Histories of HIV/AIDS in Western Europe: New and Regional Perspectives, Janet Weston and Hannah J. Elizabeth (Manchester: Manchester University Press, 2022), 192–214; Jennifer Brier, “‘I’m Still Surviving’: Oral Histories of Women Living with HIV/AIDS in Chicago,” Oral History Review 45, no. 1 (2018): 68–83; Gerald M. Oppenheimer and Ronald Bayer, Shattered Dreams : An Oral History of the South African AIDS Epidemic (Oxford: Oxford University Press, 2007); Sarah Schulman, Let the Record Show: A Political History of ACT UP New York, 1987–1993 (New York : Farrar, Straus and Giroux, 2021).

20 Healey, Russian Homophobia, 153.

21 Because of the difficulties related to accessing materials in the Russian Federation at the present moment, the article regrettably does not draw extensively on the Russophone queer press.

22 On “ad hoc archives” for researching HIV and AIDS, see Virginia Berridge, “Researching Contemporary History: AIDS,” History Workshop 38 (1994): 228–29.

23 Diane Rowland and Alexandra V. Telyukov, “Soviet Health Care from Two Perspectives,” Health Affairs (1991): 72–86.

24 Elena Knorre, “Ochnites’ liudi!” Zdorov’e 6 (1989): 19.

25 The Central Committee of the Communist Party published a response to the above article where they blamed the situation on “a number of serious shortcomings in the work of doctors and production workers who supply medical institutions” “Ot redaktsii,” Zdorov’e 6 (1989): 19. On discourses of blame, see Deborah A. Field, Private Life and Communist Morality in Khrushchev’s Russia (New York, NY, Peter Lang, 2007), 63.

26 Anne White, Democratization in Russia under Gorbachev, 1985–91: The Birth of a Voluntary Sector (London: Palgrave Macmillan, 1999), 102.

27 Stephen Lovell “Ogonek: The Crisis of a Genre,” Europe-Asia Studies 48, no. 6 (1996): 994.

28 On Ogonek during glasnost’, see Lovell “Ogonek: The Crisis of a Genre.”

29 See for example, Alla Alova, “Luchshe ne dumat’?” Ogonek, 26 (1989): 28–30; Christopher Cerf and Marina Albee ed. Small Fires: Letters from the Soviet People to Ogonyok Magazine, 1987–1990 (New York: Summit Books, 1990), 178–81.

30 Kristy Ironside, A Full-Value Ruble: The Promise of Prosperity in the Postwar Soviet Union (Cambridge, MA: Harvard University Press, 2021), 5.

31 Alena V. Ledeneva, Russia’s Economy of Favours: Blat, Networking and Informal Exchange (Cambridge: Cambridge University Press, 1998); Shelia Fitzpatrick “Blat in Stalin’s Time” in Bribery and Blat in Russia: Negotiating Reciprocity From the Middle Ages to the 1990s, ed. Stephen Lovell, Alena Ledeneva & Andre Rogachevskii (London: Macmillan, 2000), 166–82.

32 “Khronika scheta ‘Ogonek-AntiSPID,’” Ogonek 38 (1989): 17.

33 ”‘Anti-SPID’ prodolzhaet rabotat’”, Ogonek 40 (1989): 8; “Ogonek-AntiSPID: Sovetskii blagotvoritel’nyi fond,” Ogonek 52 (1990): 6. On the foundation and philosophy of the Russian Christian Democratic Movement, see Richard Sakwa, “Christian Democracy in Russia,” Religion, State and Society 20, no. 2 (1992): 138–145.

34 “‘-AntiSPID’ prodolzhaet rabotat,’” 8.

35 Hearne, “AIDS and the End of the Soviet Union.”

36 “Obrashchenie – rasporiaditel’nogo soveta Sovetskogo blagotvoritel’nogo fonda ‘Ogonek-AntiSPID’ k Verkhovnomu Sovetu SSSR i pravitel’stvu strany.”

37 Alexander, “AIDS/HIV and Homophobia,” 138.

38 Robert Hornsby, “The Enemy Within? The Komsomol and Foreign Youth inside the Post-Stalin Soviet Union, 1957–1985,” Past & Present 232, no. 1 (2016): 237–78; Anne Gorsuch, All This Is Your World: Soviet Tourism at Home and Abroad after Stalin (Oxford: Oxford University Press, 2011), 100–16.

39 The Soviet government continued to control Ogonek’s printing facilities and paper supply in this period, Gordon Baros, “Vitaly Korotich: The Press and Perestroika,” At the Harriman Institute 3, no. 3 (1990).

40 “Khronika scheta,” 17.

41 Alla Alova, “Khronika fonda ‘Ogonek-AntiSPID,’” Ogonek 2 (1990): 8.

42 On American journalists reporting from the Soviet Union throughout the 1960s–1980s, see Dina Fainberg, Cold War Correspondents: Soviet and American Reporters on the Ideological Frontlines (Baltimore: John Hopkins University Press, 2020), 184–226.

43 On religion and anti-communism, see Dianne Kirby, “Harry Truman’s Religious Legacy: The Holy Alliance, Containment and the Cold War” in Religion and the Cold War, ed. Dianne Kirby (Basingstoke: Palgrave, 2003), 77–102, and chapters 7 and 8 of David S. Foglesong, The American Mission and the ‘Evil Empire’ The Crusade for a ‘Free Russia’ Since 1881 (Cambridge: Cambridge University Press, 2007).

44 “‘AntiSPID’ prodolzhaet rabotat,’” 8.

45 Alova, “Khronika fonda,” 8.

46 Alova, “Khronika fonda,” 8.

47 “‘Ogonek-AntiSPID,’” 6.

48 “‘Ogonek-AntiSPID,’” 6.

49 “‘Ogonek-AntiSPID,’” 6.

50 “‘Ogonek-AntiSPID,’” 6.

51 “‘Ogonek-AntiSPID,’” 6.

52 This exhibition immediately became known as the Bulldozer Exhibition because the works on display were bulldozed by the KGB and the police. For more on the exhibition, see Alexander Glezer, “Soviet ‘Unofficial’ Art,” Index on Censorship 4, no. 4 (1975): 35–40.

53 “‘AntiSPID’ prodolzhaet rabotat’,” 8.

54 Alova, “Khronika fonda ‘Ogonek-AntiSPID,’” 8.

55 Holly Burkhalter, “The Politics of AIDS: Engaging Conservatives,” Foreign Affairs 83, no. 1 (2004): 8-14; Mark C. Donovan, “The Problem with Making AIDS Comfortable: Federal Policy Making and the Rhetoric of Innocence,” Journal of Homosexuality 32, no. 3–4 (1997): 115–44.

56 Jennifer Chan, Politics in the Corridor of Dying: AIDS Activism and Global Health Governance (Baltimore: John Hopkins University Press, 2015), 31–32.

57 Deborah Lupton, Moral Threats and Dangerous Desires: AIDS in the News Media (London: Taylor & Francis, 1994), 99–103.

58 Jessica Ogden and Laura Nyblade, Common at its Core: HIV-related Stigma Across Contexts (Washington DC: International Centre for Research on Women, 2005), 23.

59 N. A. Chaika, “Pervyi 40 tysiach vashikh otvetov,” Zdorov’e 5 (1990): 15; “Mir v opasnosti,” Zdorov’e 8 (1990): 13; Feshbach, “The Early Days of the HIV/AIDS Epidemic,” 30.

60 Alexander, “AIDS/HIV and Homophobia”; Siobhán Hearne, “Selling Sex Under Socialism: Prostitution in the Post-War USSR,” European Review of History 29, no. 2 (2022): 290–310.

61 Foglesong, “When the Russians Really Were Coming.”

62 Interview with Stuart Altschuler conducted on Skype, 20 March 2023. The first Citizens’ Summit was held in Washington DC in 1988. On the citizens’ summits, see the many documents on the website of Rama Jyoti Vernon, founder of the Soviet-American Centre for Dialogue, including “On The Center for Soviet-American Dialogue and the Center of International Projects” and “Twelve Joint Projects from Summit I” https://www.rama.yoga/soviet--american-summit-i.html, as well as “Citizens’ Summit II Brochure” and “Citizens’ Summitt II Summary”: https://www.rama.yoga/soviet-american-summit-ii.html (both accessed 4 October 2023).

63 Stuart Altschuler, “The International Centre for Better Health Responds to HIV/AIDS in the Former Soviet Union,” Families in Society: The Journal of Contemporary Social Services 75, no. 9 (1994): 571–73.

64 Email communication with Mitchell Kushner, 27 February 2023; Interview with Wendy Arnold conducted on Skype, 1 March 2023.

65 “VICH-infektsiia perestala byt’ ‘privilegii’ grupp riska,” Zdorov’e 8 (1990): 14–15.

66 Interview with Stuart Altschuler.

67 Petr Selinov, “Ne povtoriaite nashikh oshibok! Tete-a-tete po telefonu” SPID-Info, 1 (1991): 7.

68 Selinov, “Ne povtoriaite nashikh oshibok!” 7.

69 Official epidemiological data reported that there were only 28 cases of AIDS and 900 cases of HIV registered in the USSR by March 1990, N. A. Chaika, SPID: Eto nuzhno znat’ (St Petersburg: Ministerstvo zdravookhranenie RSFSR, 1990), 53. There was widespread scepticism about the accuracy of the figures in the press at the time.

70 Tema was established in February 1990. On the development and content of the magazine, Laurie Essig “Publicly Queer: Representations of Queer Subjects and Subjectivities in the Absence of Identity” in Consuming Russia: Popular Culture, Sex, and Society since Gorbachev, ed. Adele Marie Barker (Durham: Duke University Press, 1999), 287-88.

71 Julie Dorf and Masha Gessen, “Notes from the Revolution,” Tema International 2 (autumn 1991): 2–3.

72 In 1977, the first edition of the Frameline international LGBT film festival took place in San Francisco https://www.frameline.org/about/frameline (accessed 27 April 2023).

73 “Preservativy u sten Mossoveta,” Moskovskii Komsomolets, 31 July 1991; Tuller, Cracks in the Iron Closet, 8–9.

74 Kevin J. Gardner, “Track 1: AIDS and Health,” Tema International 2 (1991), 3.

75 Dorf and Gessen, “Notes from the Revolution,” 7.

76 Interview with Tom Boellstorff conducted on Zoom, 20 March 2023.

77 Roshchupkin discussed the goals of his hunger strike in an interview in the queer magazine 1/10, Gennadii Roshchupkin, “Ne sudite i da ne sudimy budete,” 1/10 1 (1991): 2–4.

78 The Tema programme aired on 1 December 1992 https://www.youtube.com/watch?v=l6O9LsAv7HY (accessed 1 March 2023).

79 Interview with Julie Dorf conducted on Zoom, 17 May 2023.

80 Interview with Tom Boellstorff.

81 “My i Vy,” Ty: Illiustrirovannyi zhurnal dlia gomoseksualistov i lesbiianok (1992): 18.

82 Tom Boellstorff, “Gay Activists at the Barricades,” Montage Newsletter, available at https://sites.socsci.uci.edu/~tboellst/bio/me-sent.pdf (accessed 19 March 2023).

83 For example, the Leningrad Gay Laboratory disseminated information about HIV-AIDS to gay men across the USSR in mid-to-late 1980s, Sergei P. Shcherbakov, “On the Relationship Between the Leningrad Gay Community and the Legal Authorities in the 1970s and 1980s,” Sexual Minorities and Society: Changing Attitudes toward Homosexuality in 20^th^ Century Europe ed. Udo Parikas and Teet Veispak (Tallinn, 1991), 94-105. On other transnational initiatives, see Feruza Aripova, “Fifty Shades of Vice: Decolonizing the Soviet Homophobic Legacy” Ph.D dissertation (Northeastern University, 2023), 140.

84 Interview with Julie Dorf.

85 Interview with Tom Boellstorff.

86 Interview with Julie Dorf.

87 Interview with Tom Boellstorff; Interview with Stuart Altschuler.

88 Interview with Gennady Roshchupkin.

89 Interview with Julie Dorf; Dorf and Gessen, “Notes from the Revolution.”

90 Tamar Shirinian, “Querying Identity: Misrecognition as Performative Refusal in Armenian LGBT Advocacy” in Decolonizing Queer Experience: LGBT+ Narratives from Eastern Europe and Eurasia, edited by Emily Channell-Justice (Lanham: Lexington Books, 2020), 41.

91 Interview with Julie Dorf.

92 Shirinian, “Querying Identity,” 39. On the NGOization of LGBT activism in the 1990s more broadly, see David Paternotte, “The NGOization of LGBT Activism: ILGA-Europe and the Treaty of Amsterdam,” Social Movement Studies 15, no. 4 (2016): 388–402.

